# The* In Vitro* Lipolysis of Lipid-Based Drug Delivery Systems: A Newly Identified Relationship between Drug Release and Liquid Crystalline Phase

**DOI:** 10.1155/2016/2364317

**Published:** 2016-05-16

**Authors:** Lu Xiao, Tao Yi, Ying Liu, Hua Zhou

**Affiliations:** ^1^State Key Laboratory of Quality Research in Chinese Medicine, Macau Institute for Applied Research in Medicine and Health, Macau University of Science and Technology, Macau; ^2^School of Health Sciences, Macao Polytechnic Institute, Macau; ^3^Pharmacy Department, Wuhan Medical Treatment Center, Wuhan, China

## Abstract

The purpose of this study was to offer a new insight into the microstructure changes during* in vitro* lipolysis of five lipid-based drug delivery formulations belonging to different lipid formulation types. Five lipid-based formulations of indomethacin were investigated using an* in vitro* lipolysis model. During lipolysis, microstructures of the intermediate phase formed by lipolytic products were observed. The results showed that the time of liquid crystal formation during* in vitro* digestion for these formulations was Type I > Type II > Type IIIB > Type IV > Type IIIA (*p* < 0.05). After lipolysis, the drug releases from these formulations were determined. The results showed that the amount of drug distributed in the aqueous phase, obtained by ultracentrifuge after lipolysis, was, astonishingly, in inverse rank order of the above mentioned, that is, Type IIIA > Type IV > Type IIIB > Type II > Type I (*p* < 0.05). These results showed that the liquid crystalline phase probably has a critical influence on the fate of the drug during* in vitro* lipolysis and suggested that the liquid crystalline phase facilitated drug precipitation. These findings may improve the understanding of lipolysis of lipid-based drug delivery systems for designing better delivery system.

## 1. Introduction

The use of lipid-based formulations as a strategy for enhancing the oral bioavailability of lipophilic drugs has engendered much scientific and commercial interest. Lipid carriers, which can be formulated as lipid solutions, lipid suspensions, emulsions, microemulsions, and self-emulsifying systems, provide versatility for drug delivery. A classification system for lipid formulations was proposed by Pouton in 2000 and modified in 2006 [1, 2]. This classification system helps to identify lipid formulations and offers a guideline for comparing different formulations and data from different laboratories. According to the lipid classification system [1, 2], Type I formulations are formulations encapsulating a drug in 100% oils (triglycerides or mixed glycerides), where digestion is required for drug release. Type II formulations are water-insoluble self-emulsifying drug delivery systems encapsulating drug in 40–80% oils and 20–60% water-insoluble surfactants. Type III systems are self-emulsifying or self-microemulsifying drug delivery systems. Type IIIA formulations contain 40–80% oils and 20–40% water-soluble surfactants as well as 0–40% hydrophilic cosolvents. Type IIIB formulations contain a greater proportion of water-soluble components (20–50% water-soluble surfactants and 20–50% hydrophilic cosolvents) and a lesser proportion of oils (<20%). Type IV formulations contain mostly hydrophilic surfactants and cosolvents and represent the most hydrophilic formulations, containing no oils. Type IV formulations encapsulate drug in 30–80% water-soluble surfactants and 0–20% water-insoluble surfactants as well as 0–50% hydrophilic cosolvents.

Improvement of oral bioavailability may occur via several mechanisms, the most important of which is an increase in gastrointestinal solubilization [3]. For lipid-based delivery systems, performance is governed by the fate of the delivery system in the gastrointestinal tract rather than by particle size in the initial dispersion [4]. This indicates that particle size differences between Types I, II, III, and IV formulations might not be the main reason for their different behaviors* in vivo*. The drug dissolution in the gastrointestinal tract is of great interest. When drug precipitation occurs, the lipid formulation may no longer be superior to the free drug.

In recent years, the* in vitro* lipolysis model has provided a great simulation of the* in vivo* lipid digestion process. This* in vitro* model had been utilized to assess lipid-based delivery systems, improving our understanding of drug solubilization and release. Different lipolysis digestion phases can be separated by ultracentrifugation. The aqueous phase, which contains colloidal phases and drug dissolved in the aqueous phase, is a prerequisite for absorption. The concentration of drug in the aqueous phase has great significance for absorption. The sediment phase contains calcium soaps of fatty acids and precipitated drug. It is believed that the amount of the solid, precipitated drug, and the redissolution rate influence absorption [5]. To further clarify the mechanism of lipid digestion, the microstructures formed during digestion have been studied [6]. Recent studies visualizing lipid digestion have identified a relationship between intermediate phases and drug solubilization [7, 8]. The intermediate phases play an important role in the entire performance of the lipid formulation in the gastrointestinal tract [8, 9]. However, it is still not clear how the intermediate phases act on lipid-based formulations during digestion. It is also not clear how differences among different lipid formulations affect lipid lipolysis. The mechanisms of lipid lipolysis* in vivo* may be complicated, perplexing, and confusing. Describing the course of drug trafficking and lipolysis more completely would help elucidate the mechanism of digestion of lipid-based formulations in the gastrointestinal tract.

The aim of the current study was to understand the differences in lipolysis of different formulations belonging to different lipid formulation types. Using the* in vitro* lipolysis model, this study observed microstructures of the intermediate phase formed by lipolytic products and investigated the drug release and solubilization of different lipid-based drug formulations after digestion. An interesting relationship between microstructure changes (especially the liquid crystal structure formed during lipolysis) and drug release was found, indicating that absorption occurred* in vivo*.

## 2. Material and Methods

### 2.1. Material

Trizma maleate 99.5%, sodium taurodeoxycholate 97% (NaTDC) and porcine pancreatin (8× USP specifications) were purchased from Sigma chemical company (St. Louis, MO, USA). 4-BPB (4-bromophenylboronic) acid was purchased from Sigma-Aldrich chemical company (St. Louis, MO, USA). Powdered lecithin (approximately 80% pure PC, from egg yolk) was purchased from A.T.V. pharmaceutical technology company (Shanghai, China). Polyoxyl 35 castor oil (Cremophor® EL) was obtained from BASF Corp. (Mount Olive, NJ). Mono-/diglyceride of medium chain fatty acids (mainly caprylic and capric) (Capmul® MCM) and medium-chain-length triglyceride consisting of 57.4% w/w caprylic acid (C8), 42.1% capric acid (C10), and 0.4% lauric acid (C12) were obtained from Abitec Corp. (Janesville, WI). Saturated polyglycolysed C6–C14 glycerides (Labrasol®) and diethylene glycol monoethyl ether (Transcutol® P) were obtained from Gattefossé Corp. (Lyon, France). Tween 85 was purchased from local suppliers. Indomethacin was purchased from Zizhu Pharmaceutical Corp. (Beijing, China, insoluble in water 0.94 mg/L). Other chemicals were of HPLC or of analytical grade.

### 2.2. Lipid Formulation Preparation

The compositions of the lipid-based formulations listed in Table 1 were selected after preliminary experiments to determine the optimal conditions for each lipid formulation. A known composition (containing drug at 50% of the saturated solubility for each formulation) was mixed and stirred in a water bath at 40°C until a transparent and homogeneous lipid solution was formed to create each lipid formulation.

### 2.3. Solubility Study

The equilibrium solubility of indomethacin was determined in Type I, Type II, Type IIIA, Type IIIB, and Type IV lipid formulations and in the, respectively, blank aqueous phase obtained by digesting 250 mg of each lipid formulation for 30 min at 37°C. The equilibrium solubility of indomethacin in digestion buffer, NaTDC/PC, and formulations with NaTDC/PC were also determined under the same conditions. The excess drug was added to a volume of the lipid formulations and the drug was dissolved by vortex mixing. The mixture was continuously stirred at 37°C for 48 h. After equilibration the suspension was separated by centrifugation and the solution was analyzed by HPLC. The determination was performed in triplicate and the average value was used.

### 2.4. Particle Size Measurement

The average droplet size and polydispersity index of microemulsions from Type I, Type II, Type IIIA, Type IIIB, and Type IV lipid formulations were assessed by photon correlation spectroscopy analysis (Nano ZS90, Malvern Instruments, UK) at a scattering angle of 90° at 25°C. An equivalent amount of each formulation was added to equivalent volumes of distilled water. The determination was performed in triplicate and the average value was used.

### 2.5. *In Vitro* Lipolysis

An* in vitro* lipolysis model employed to characterize the lipid-based formulations in the intestines was conducted as previously described [10, 11]. Briefly, the experimental set-up consisted of a thermostable (37°C) reaction vessel under continuous agitation (100 rpm) and a pH-stat (pH 7.5) with an autoburette utilized to add a 0.2 M NaOH solution during lipolysis. The number of OH^−^ ions present in the volume of the titrant undergoing lipid digestion could be equated with the amount of free fatty acid liberation caused by lipolysis.

The experimental medium, which simulated the fed state in the gastrointestinal tract, composed 9 mL of digestion buffer (50 mM Trizma-maleate, 150 mM NaCl, 5 mM CaCl_2_·2H_2_O, pH = 7.5) containing 20 mM NaTDC and 5 mM PC; the medium was continuously stirred at 37°C. 250 mg of each tested lipid formulation (containing drug at 50% of the saturated solubility of each respective formulation) was dispersed in the medium and stirred for 15 min at pH 7.5. 1 mL of a pancreatin extract (containing 10,000 TBU of pancreatic lipase activity) was added to initiate the digestion experiment [10]. The digestion experiments were maintained at pH 7.5 using a pH-stat. The experiments were conducted over 30 min before adding 4-BPB to terminate the experiment [11, 12]. After lipolysis, 10 mL of the postdigestion mixture was ultracentrifuged (334,000 g, 30 min, 16°C, Cp 100MX preparative ultracentrifuge, P80AT rotor, Hitachi Koki Ltd., Tokyo, Japan) to achieve separation [10, 11].

### 2.6. HPLC-Analysis

Samples from the solubility study and* in vitro* lipolysis study were tested to measure the content of indomethacin by HPLC (Agilent 1200 series, Agilent, USA). A C18 column (Dikma, 5 *μ*m, 4.6 mm ID × 25 cm) was used. The mobile phase consisted of methanol and 0.4% glacial acetic acid (80 : 20 v/v) and the flow rate was 1.0 mL/min. A 10-*μ*L aliquot of the sample was injected directly into the HPLC, and the effluents were monitored at 320 nm.

### 2.7. Optical Microscopy

The samples were withdrawn from the lipolysis medium at specific time points (0, 1.5, 3.5, 5, 10, and 30 min) and observed under an optical microscope. A droplet of the samples was placed on a microscope slide and covered with a cover slip. The microstructure of the samples was determined using light microscopy, in order to detect the sequence of events that occur during lipid formulation digestion [13].

### 2.8. Statistical Analysis

All results were expressed as mean ± SD. The data from different formulations were compared for statistical significance by one-way analysis of variance (ANOVA).

## 3. Results and Discussion

### 3.1. Properties of Formulations

This study selected five formulations belonging to different formulation types mentioned above. The content of the formulations shown in Table 1 demonstrates that the selected formulations exactly conform to each lipid formulation classification. The average diameters of the lipid droplets formed upon dilution of the five different formulation types in water were assessed by correlation spectroscopy (Nano ZS90, Malvern Instruments, UK) at a scattering angle of 90° at 25°C. The size, polydispersity index, and equilibrium solubility of indomethacin for each lipid formulation is shown in Table 2. The average diameter of each dispersion also conforms to the typical particle size for each lipid formulation classification [1]. Thus, the selected formulations fit the requirements for each type of lipid formulation. The solubility of indomethacin directed the drug loading level used to prepare the lipid solution. The drug was dissolved at 50% of its saturated solubility in each formulation. Because the process of lipid lipolysis is complicated, the composition and properties of the lipid carriers may significantly impact the lipolysis of the formulations. The primary purpose for selecting formulations belonging to the different lipid formulation types was twofold. First, it allowed us to describe the common rules of each lipid classification. Moreover, it allowed us to use the diversity between classifications to gain new understanding about differences in lipolysis between classifications.

### 3.2. Lipolysis Study and Drug Release

**Figure 1:**
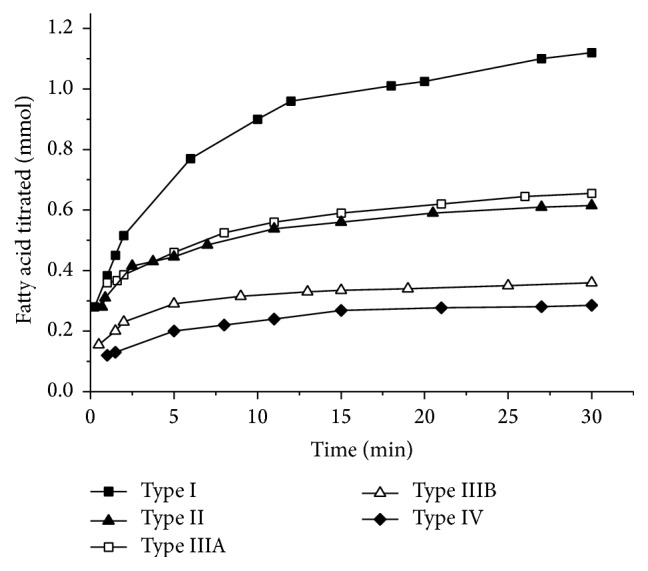
The amount of fatty acids titrated during a 30 min* in vitro* digestion period under simulated fed state for the different lipid formulations (*n* = 5).

**Figure 2:**
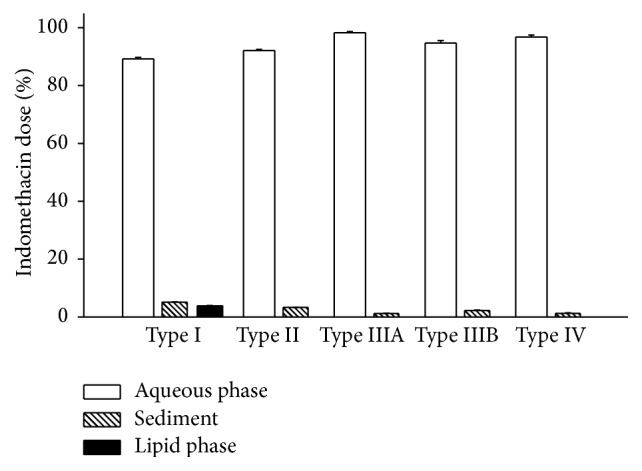
Release of indomethacin across the aqueous phase, sediment and lipid phase of the* in vitro* lipolysis model resulting from the different lipid formulations. The data are presented as the mean ± SD, with *n* = 5 for each formulation.

An established* in vitro* lipolysis model, conducted as above described, was employed to characterize the lipid-based formulations in intestine. The lipolysis profiles of Type I, Type II, Type IIIA, Type IIIB, and Type IV formulations were presented in Figure 1. The release of indomethacin across the different phases of the* in vitro* lipolysis model resulting from Type I, Type II, Type IIIA, Type IIIB, and Type IV formulations was presented in Figure 2. According to the data, the amount of total indomethacin dissolved in the aqueous phase was Type IIIA > Type IV > Type IIIB > Type II > Type I. However, this did not correlate with drug precipitation upon dispersion. The likelihood of drug precipitation upon dispersion is negligible for Types I and II, where the quantities of relatively lipophilic components are high. The likelihood of drug precipitation on dispersion increases for Types III and IV, which contain large quantities of surfactants and cosurfactants [3]. Therefore, drug precipitation upon dispersion might not closely correlate with drug release. The nature of the formulations might be the reason for the lack of conformity. More importantly, lipolysis might have multiple factors that impact the degradation products, fatty acids, bile salts, and phospholipids. The most important factor influencing drug release still needs to be determined. This question is further investigated in studies conducted to complete phase analysis of lipid system during digestion [8, 9, 14–16], which demonstrate that a liquid crystalline phase occurred in the process of lipid lipolysis. However, a clear explanation of this mechanism and process has not yet been demonstrated.

### 3.3. Equilibrium Solubility in Digestion Medium and Blank Aqueous Phases from Resulting Digests

Lipid-based formulations improve oral bioavailability via several possible mechanisms and the most important point is the enhancement of drug solubility in the gastrointestinal tract [3]. The equilibrium solubility of Indomethacin in digestion buffer, NaTDC/PC, and the blank aqueous phase obtained by the* in vitro* lipolysis medium with formulations after 30 min's lipolysis were presented in Table 3. For five formulations, lipolysis substantially improved drug solubility compared with the drug in respective lipid formulation, the digestion buffer, and NaTDC/PC. The rank order of solubility of indomethacin in these blank aqueous phases was Type I > Type II > Type IIIA > Type IIIB > Type IV. This data suggested that, after lipolysis, solubility of the drug in formulations composed with large percentage of triglyceride (fat) would likely be higher whereas in formulation composed with more either surfactant or cosurfactant which might offer less help for enhancing solubility in aqueous phase. Triglyceride digestion products such as monoacylglycerides and fatty acids were available for the building of mixed micelles which were necessary for solubilizing poorly water-soluble drugs [17]. This was in accordance with some study on simple triglyceride lipid formulations which demonstrated that substantial improvements in solubility could be attained in the presence of a digesting lipid [12].

The solubility of indomethacin in the blank aqueous phases from resulting digests of each selected formulation was remarkably higher compared to each formulation in digestion medium before lipolysis (*p* < 0.001), except Type IV (*p* > 0.05) (Table 3). The solubilization of drug attributes to micellar solubilization [18]. Kossena et al. presented that, during lipid digestion, a range of vesicular and micellar species with endogenous bile salts and phospholipids formed and lipidic digestion products enhanced solubilization [19]. Under digestion condition, the data showed that, not as other types of lipid formulations, type IV did not obtain solubilization by lipolysis. This might be because Type IV formulation did not contain natural lipids and represent the most hydrophilic formulations for the sake of offering increased drug loads (due to higher drug solubility in the surfactants and co-solvents) [1, 3]. The formulations comprising the high concentration or proportion of surfactants would be less effective in maintaining drug solubilization [20]. Moreover, literatures also showed that the digestion of the surfactants may cause the precipitation of the poorly water soluble drugs and therefore limit oral bioavailability [17]. However, there is limited knowledge of the complex mechanisms and interactions between degradation products and endogenous lipids. In order to understand more about the process of lipid digestion and therein interdependencies and mechanisms of solubilization of digestion products, internal changes of lipid formulation during digestion should be investigated. To further understand the drug solubilization and release of the five formulations, the formation of intermediate phase of lipid-based drug delivery systems was observed.

### 3.4. Optical Microscopy Study

During lipid digestion, several factors controlled drug trafficking. Many of these factors are unknown but may be related to the nature of the drug and its affinity for the various intermediate digestion phases [8, 21]. Recent research demonstrated that the intermediate phases produced during lipid digestion have a significant impact on drug solubilization and lipid digestion [10–12], thereby influencing the overall performance of the drug system in the gastrointestinal tract [8]. Under simulated physiological conditions, fat digestion can be observed using optical microscopy [22]. During the process of fat digestion, a sequence of physicochemical events occurs, producing phases visible by light microscopy. Even under physiological conditions, the phases are not easily dispersed by bile salts, enabling facile observation.

**Figure 3:**
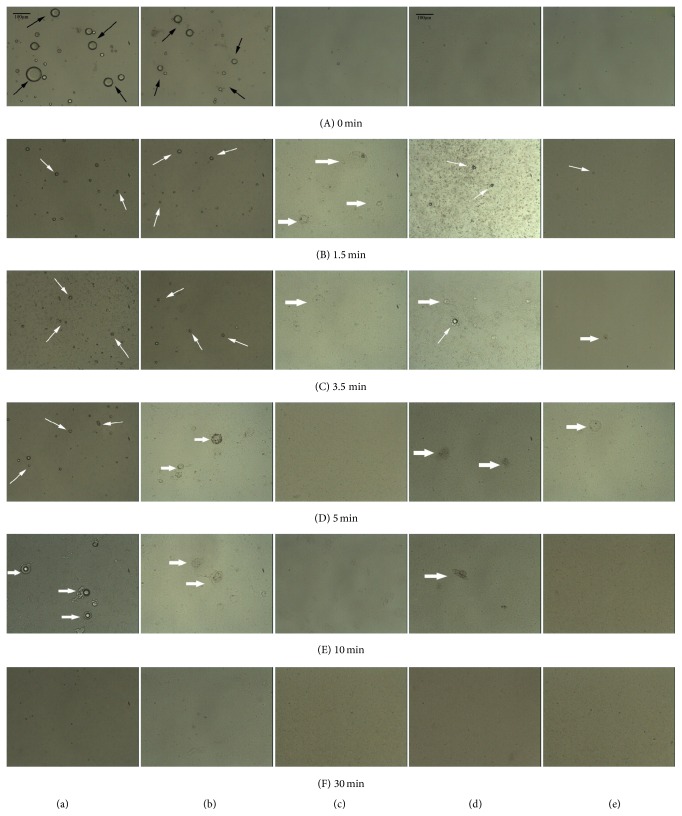
The sequence of events that occur during (a) Type I, (b) Type II, (c) Type IIIA, (d) Type IIIB, and (e) Type IV lipid formulation lipolysis, respectively, has been observed via microscope (black arrow: lipid droplet; white arrow: liquid crystalline; and thick arrow: viscous isotropic). The photomicrographs ((a) to (e)) were taken from the same reaction sequence at 0, 1.5, 3.5, 5, 10, and 30 minutes, respectively. Scale bars (black line in (a)(A) and (d)(A)), 100 *μ*m.

In this study, microstructures formed during the digestion of the five formulations. Each of the formulations, which were observed by optical microscopy, is representative of a different type of lipid system. For all of the formulations, there was a clear decrease in the number of lipid droplets over time; this decrease can be attributed to digestion and solubilization of the oil within mixed micelles. The timed photographic sequence shown in Figures 3(A)–3(F) was taken during the enzymatic reaction of a single formulation. Intact lipid droplets of Type I were shown in Figure 3(A) before the addition of enzyme. When the lipase initiates lipid digestion, the surface of the lipid droplets presented crenate. Crenation occurred within the first 5 minutes, as shown in Figures 3(a)(B)–3(a)(D). Crenation was considered the first liquid crystalline product phase [22]. During lipid digestion, the second “viscous isotropic” phase formed from the remaining lipid droplets. Figures 3(a)(E) and 3(a)(F) showed that the viscous isotropic phase increases as lipid droplets diminish in size. A similar phenomenon was observed in Type II, Type IIIB, and Type IV formulations, except that the liquid crystalline phase appeared at different times for different formulations. Evidence of liquid crystalline and viscous isotropic phase formation can be seen in Figure 3. During lipid digestion, both the liquid crystalline and viscous isotropic phases can be observed in Type I, Type II, Type IIIB, and Type IV formulations whereas for Type IIIA formulations only the viscous isotropic phase can be observed.

Liquid crystalline structures are formed by the interaction of lipid digestion products with endogenous surfactants such as bile salts and phospholipids which help to reduce precipitation and maintain dissolution of the drug [23]. The factors influencing both drug release and the formation of the liquid crystalline phase may be not only the nature and quantity of the formulations but also the concentration of endogenous bile salts [24]. Porter's investigation showed that bile duct ligation was performed to prevent digestion from affecting the phase structure [8], which is in agreement with the observation that bile salts are necessary for phase structure formation during lipid digestion [9, 21, 25].

Furthermore, as shown in Figure 3, the appearances of the liquid crystalline phase in Type I, Type II, Type IIIB, and Type IV formulations were at 1.5–5 min, 1.5–3.5 min, 1.5–3.5 min, and 1.5 min, respectively. In Patton's study, researchers observed thickening of the liquid crystalline before it reached a critical stage in which the shell cracked and unhydrolyzed lipid was expelled. The fact that the formation of lipid phases during digestion is a rapid process has also been previously demonstrated in other studies utilizing light microscopy, where lamellar structures formed within 1.5 min of the initiation of the reaction and liquid crystalline appeared during 1.5 min to 3.5 min [22, 26]. In our comparison of the five different formulation types, the quantity of formulations for lipolysis was larger than that in the study mentioned above, which may cause the lipids to exist for a longer period of time.

### 3.5. Relationship between Liquid Crystalline Formation and Drug Release

Visualizing investigations of lipid digestion have demonstrated that various phases were produced during lipolysis; these intermediate phases played an important role in the performance of the formulation. During lipid lipolysis, the mixed micelles are expected to carry the drug to the unstirred water layer lining the intestine, release the drug, and thereby facilitate the absorption [5]. There might be a relationship between microscopic structures of intermediate phases and drug trafficking and absorption. In our study, considering that the amount of the liquid crystalline phase in Type II was greater than the amount of the liquid crystalline phase in Type IIIB, the amount of liquid crystalline formation was Type I > Type II > Type IIIB > Type IV > Type IIIA. This order is opposite of the order of the formulations with respect to the amount of drug release in the aqueous phase. For these five formulations belonging to different lipid formulation types, the more liquid crystalline formed, the more drug distributed in the aqueous phase and the less drug precipitated in sediment phase. It suggests that the formation of the liquid crystalline phases may be an important factor influencing drug release. Lipolysis is initiated at the surface of the triglyceride droplets and causes digestion products to form liquid crystalline structures. In the presence of sufficient bile salt concentrations, the liquid crystalline phase developed both multilamellar and unilamellar vesicles [25]. Considering that the formation of the liquid crystalline structure occurred at the surface of droplets, it was supposed that the liquid crystalline structure restrained lipolysis and was therefore unfavorable to the release of the drug in the aqueous phase. Another explanation may be that the ordered structure of the liquid crystalline phase has the tendency to form drug deposition. In a word, there has been a possible relationship between drug release and liquid crystalline formed during the* in vitro* lipolysis of lipid-based drug delivery systems.

**Figure 4:**
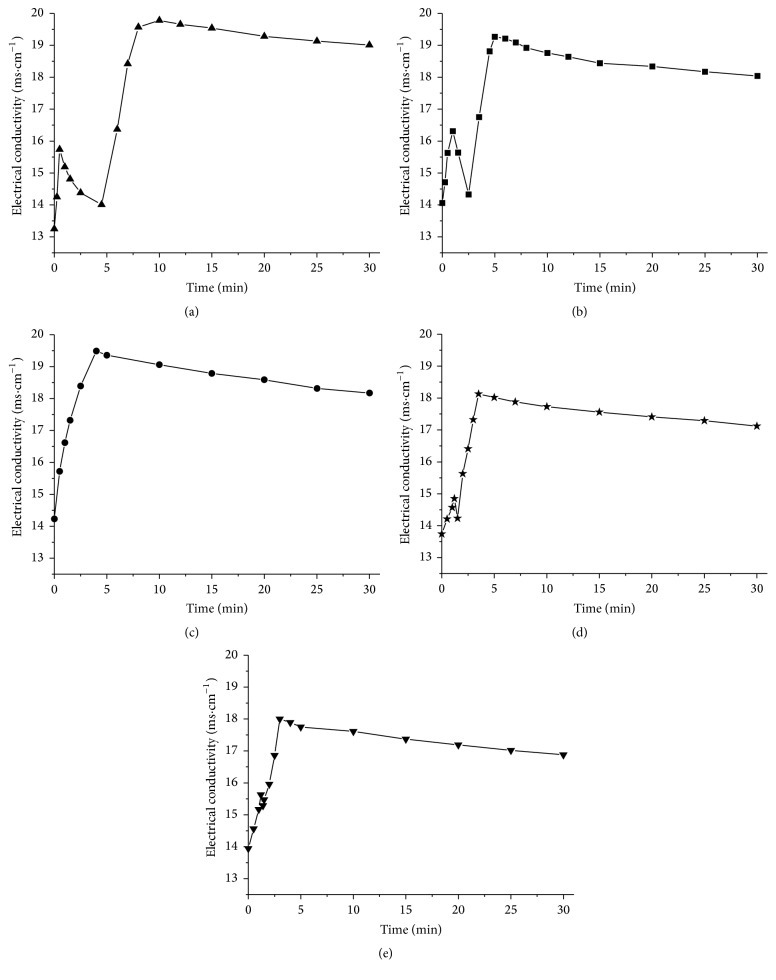
Changes in electrical conductivity of the different lipid formulations over the* in vitro* digestion period (*n* = 3). The determination was performed in triplicate and the average value was used. (a) Type I, (b) Type II, (c) Type IIIA, (d) Type IIIB, and (e) Type IV.

Some other data may increase above possibility. The conductivity of microemulsion was used to respond to the effects of the solubilized drug on the microstructure of microemulsion and structural transitions occurring upon dilution with aqueous phase [27–29]. The electrical properties of lipolytic systems had been detected to identify the state of the intermediate colloidal phases formed during lipolysis and indicate changes in these phases. Electrical conductivities of the different lipid formulations were measured throughout the course of the* in vitro* digestion, providing information about the quantity and intensity of charged particles in the lipolytic systems. The relationship between electrical conductivity value and time was illustrated in Figure 4. The results demonstrated that continuous liberation of free fatty acids throughout lipolysis caused NaOH solution to be titrated into the lipolytic system, leading to a rise in electrical conductivity. As a result, all five formulations had a rapid increase in electrical conductivity in the first few minutes. Following this initial rise period, all of the formulations except for Type IIIA showed a transitory fluctuation (decrease and then increase) in electrical conductivity before the last slow descent period (Figures 4(a), 4(b), 4(d), and 4(e)). On the contrary, the electrical conductivity of Type IIIA decreased slowly after the initial rise period (Figure 4(c)).

Data from Types I, II, IIIAB, and IV formulations showed an interesting pattern. The duration of the fluctuation in electrical conductivity was different for each formulation. The period of time over which there was a fluctuation in electrical conductivity was Type I > Type II > Type IIIB > Type IV > Type IIIA, where the time period of fluctuant electrical conductivity for Type IIIA as 0 min. This order is identical to the order of the amount of liquid crystalline formation. A previous study by Patton showed that hydrolysis of emulsified fat droplets by pancreatic lipase in the presence of colipase and bile salt micelles generated the lamellar liquid crystalline phase, the viscous isotropic phase, and the crystalline phase; crystalline phases contain calcium and ionized fatty acid [22]. Therefore, a decrease in electrical conductivity following the initial rise period was likely due to the formation of the liquid crystalline phase, which reduced ion concentrations. According to the above data, the amount of total drug in the sediment phase was Type I > Type II > Type IIIB > Type IV > Type IIIA (this order is identical to the order of the duration and the amount of liquid crystalline formation). Consequently, these results further suggest the possibility that the liquid crystalline phase is a critical factor for the fate of drugs and facilitates drug precipitation of lipid-based drug delivery systems.

## 4. Conclusions

Based on an* in vitro* lipolysis model, we investigated drug solubilization and drug release after lipid digestion and observed the colloidal structures generated during lipolysis by light microscope. The data indicated that performance decreases with liquid crystalline formation (Type I > Type II > Type IIIB > Type IV > Type IIIA) and increases in drug release in aqueous phase (Type IIIA > Type IV > Type IIIB > Type II > Type I). The results suggest that liquid crystals formed during lipid digestion may be a factor unfavorable for absorption. Using light microscope to observe microstructure formation during lipid digestion, we found the possible relationship between drug release and liquid crystalline formed during the* in vitro* lipolysis of lipid-based drug delivery systems. These results may improve the understanding of the mechanism behind intestinal lipid digestion and absorption of lipid-based formulations.

## Figures and Tables

**Table 1:** Composition (%, w/w) of the lipid formulations.

Lipid excipients	Type I	Type II	Type IIIA	Type IIIB	Type IV
Capmul MCM	100.0	50.0	50.0	15.0	—
Cremophor EL	—	—	15.0	15.0	30.0
Labrasol	—	—	15.0	15.0	30.0
Tween 85	—	50	—	—	15.0
Transcutol P	—	—	20.0	55.0	25.0

**Table 2:** Equilibrium solubility at 37°C, the particle size of dispersion, and polydispersity index of the lipid formulation (*n* = 3, the average value was used).

Type	Equilibrium solubility of indomethacin in lipid formulation (mg·g^−1^)	*Z*-Ave (nm)	PDI
Type I	12.17	coarse	N/A
Type II	32.19	130.20	0.28
Type IIIA	68.89	57.14	0.33
Type IIIB	108.60	14.55	0.20
Type IV	100.39	12.21	0.19

**Table 3:** Equilibrium solubilities at 37°C of indomethacin in digestion buffer pH 7.5, in NaTDC/PC and in blank aqueous phases obtained from drug-free lipid formulations digest (simulated fed state) (*x* ± *s*, *n* = 3).

Medium	Equilibrium solubilities 37°C (*μ*g·mL^−1^)
Digestion buffer	299.73 ± 22.15
NaTDC/PC	2009.55 ± 45.92
Type I + NaTDC/PC	2192.26 ± 12.33
Type II + NaTDC/PC	2336.14 ± 97.67
Type IIIA + NaTDC/PC	2547.14 ± 54.65
Type IIIB + NaTDC/PC	2758.14 ± 61.37
Type IV + NaTDC/PC	2901.14 ± 35.86
Aqueous phase from lipid formulation digested Type I	4702.30 ± 323.21^*∗∗∗*^
Aqueous phase from lipid formulation digested Type II	4355.77 ± 28.62^*∗∗∗*^
Aqueous phase from lipid formulation digested Type IIIA	3587.67 ± 78.50^*∗∗∗*^
Aqueous phase from lipid formulation digested Type IIIB	3240.28 ± 243.94^*∗∗∗*^
Aqueous phase from lipid formulation digested Type IV	2951.54 ± 22.54

^*∗∗∗*^ *p* < 0.001 versus NaTDC/PC, Type I + NaTDC/PC, Type II + NaTDC/PC, Type IIIA + NaTDC/PC, and Type IIIB + NaTDC/PC.
